# Smart Flood Detection with AI and Blockchain Integration in Saudi Arabia Using Drones

**DOI:** 10.3390/s23115148

**Published:** 2023-05-28

**Authors:** Albandari Alsumayt, Nahla El-Haggar, Lobna Amouri, Zeyad M. Alfawaer, Sumayh S. Aljameel

**Affiliations:** 1 Computer Science Department, Applied College, Imam Abdulrahman Bin Faisal University, P.O. Box 1982, Dammam 31441, Saudi Arabia; 2 Saudi Aramco Cybersecurity Chair, Department of Computer Science, College of Computer Science and Information Technology, Imam Abdulrahman Bin Faisal University, P.O. Box 1982, Dammam 31441, Saudi Arabia

**Keywords:** UAVs, IPFS, FDSS, DeepAL, PHE, SGD, IoT, IoD, MEC, blockchain, FDSS, homomorphic, AI

## Abstract

Global warming and climate change are responsible for many disasters. Floods pose a serious risk and require immediate management and strategies for optimal response times. Technology can respond in place of humans in emergencies by providing information. As one of these emerging artificial intelligence (AI) technologies, drones are controlled in their amended systems by unmanned aerial vehicles (UAVs). In this study, we propose a secure method of flood detection in Saudi Arabia using a Flood Detection Secure System (FDSS) based on deep active learning (DeepAL) based classification model in federated learning to minimize communication costs and maximize global learning accuracy. We use blockchain-based federated learning and partially homomorphic encryption (PHE) for privacy protection and stochastic gradient descent (SGD) to share optimal solutions. InterPlanetary File System (IPFS) addresses issues with limited block storage and issues posed by high gradients of information transmitted in blockchains. In addition to enhancing security, FDSS can prevent malicious users from compromising or altering data. Utilizing images and IoT data, FDSS can train local models that detect and monitor floods. A homomorphic encryption technique is used to encrypt each locally trained model and gradient to achieve ciphertext-level model aggregation and model filtering, which ensures that the local models can be verified while maintaining privacy. The proposed FDSS enabled us to estimate the flooded areas and track the rapid changes in dam water levels to gauge the flood threat. The proposed methodology is straightforward, easily adaptable, and offers recommendations for Saudi Arabian decision-makers and local administrators to address the growing danger of flooding. This study concludes with a discussion of the proposed method and its challenges in managing floods in remote regions using artificial intelligence and blockchain technology.

## 1. Introduction

**Figure 1:**
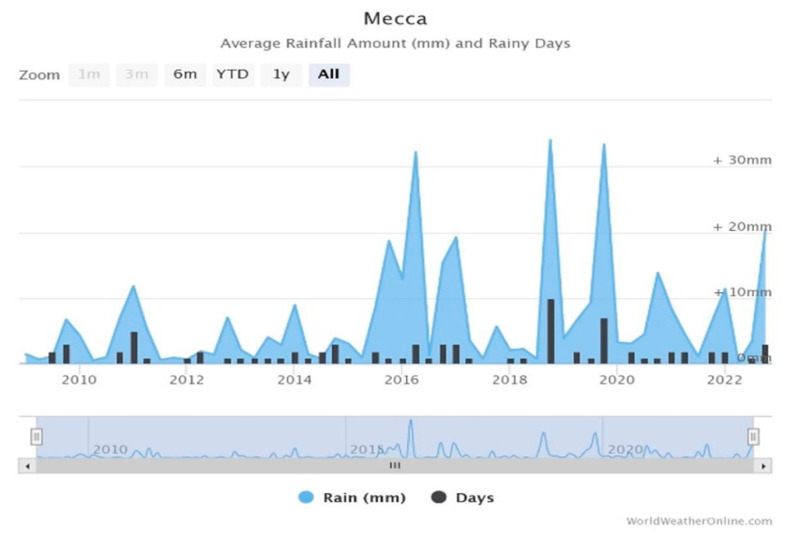
Mecca Annual Average Rainfall (worldweatheronline.com (accessed on 25 May 2023)).

Climate change continues to have an increasing impact on the planet. As a result, cities and other locations worldwide have experienced an increase in extreme weather events such as rainfall, resulting in increased flooding that has damaged private and public property, reducing insurance coverage. Mecca has been impacted by climate change in the last decade as floods have increased despite the city’s location in the Arabian Gulf, which has a hot and wet climate. According to the General Authority for Statistics in Mecca, curve flood modeling is used to analyze the volume and peak of rainfall between 2010 and 2022. Since 2010, the average peak rainfall has increased by 350%. Mecca experienced torrential rains on 23 December 2022, at least partly because of its location, surrounded by mountains, causing numerous vehicles to be swept away. Figure 1 shows Mecca’s annual average rainfall.

There are many uses for UAVs, commonly known as drones, in the commercial, agricultural, and industrial industries, such as disaster relief, power inspections, and express transportation [1]. They have adjustable altitude, high mobility, flexibility, and radio frequency communication links to ground users, so they avoid obstacles and reduce shadowing. Emergency situations and remote locations where wired infrastructure is not available usually use UAV-based wireless communications. In addition, small UAVs are energy- and economically efficient and can be used by mobile ground users dispersed throughout a broad region. Various classification criteria are available to UAVs based on their applications and targets, especially in communication scenarios, hardware, and altitude. In communications scenarios [2], UAVs perform three main functions:

- UAV-based aerial base stations (UABSs) provide network, uplink, and downlink communications for users on the ground, such as in disaster relief.
- UAV-based aerial users provide consistent and low-latency connectivity, including real-time video streaming, using UAVs as flying mobile users.
- UAV-based wireless rerouting enhances a coverage area.

UABS have been widely studied in recent years compared to ground fixed-base stations because they can reduce network communication pressure and respond quickly to ground-user needs. A single UABS, however, is often unable to meet the demand for multi-application scenarios because of the high demand for wireless services. A multi-UABS network could solve this issue, but there are three main challenges to overcome: interference management, trajectory planning, and network topology. In UAV hardware, there are two main groups of UAVs. The fixed-wing UAV is a small airplane with fixed wings that moves forward to remain airborne because of its high speed and heavy weight. Rotary-wing UAVs have non-stationary wings, and restricted mobility and weight, which allows them to remain motionless in the air and move freely in all directions. Flying altitude [1] categorizes UAVs into two groups. High altitude platforms (HAPs) fly up to 17 km above the Earth’s surface and remain motionless; they are designed for long-term applications, as they can fly for months while wirelessly covering a large geographic area. However, they are expensive and rarely deployed. Compared to HAPs, low-altitude platforms can fly higher and have better mobility. They can be used in emergency situations and unforeseen circumstances.

UAV limitations include the lack of a line of sight between the controller and the device and an inability to fly in bad weather [3]. The main challenges with UAVs are processing and managing massive amounts of data, especially when using artificial intelligence to extract and utilize valuable information. Their limited battery capacity and computational power prevent the execution of complicated algorithms. The European Telecommunications Standards Institute (ETSI) proposed mobile edge computing (MEC) in 2014 [4]. During the COVID-19 pandemic, UAV MEC architectures were proposed to integrate UAVs into MEC networks to share personal data with external parties for intelligent medical analytics. However, this raised privacy concerns [5]. One of MEC’s significant advantages is that it can be provided by UAVs from a ground station or IoT device. In the cloud, this reduces latency issues and difficulties caused by low throughput and single-node failures.

However, various threats exist, such as man-in-the-middle and spoofing attacks. Data collection can be challenging, and stored data may be altered without authorization, raising concerns about their integrity. UAV-supported MEC networks require a distributed learning strategy that enables modeling without publishing raw data [5,6]. In natural disasters, UAVs’ real-time data acquisition can prevent harm by controlling operations efficiently. They can be used to obtain aerial photographs and read water levels, wind speeds, and water speeds to predict weather events, prevent disasters, and aid rescues. To exchange information such as locations, directions, activities, and objectives, multi-drone operations require secure interactions between themselves and the MEC. These complex interactions can be achieved using AI, the computer-based system that executes tasks requiring intelligence. Among the various forms of artificial intelligence, we discuss machine learning (ML), in which training data and other information are used to form predictive models. In machine learning, the model’s predictive ability increases as the amount of training data grows. The idea of utilizing ML and AI in security and privacy is not new, but the development of deep learning algorithms has heightened their importance. Most techniques have been devoted to simulating attack patterns with specific, brittle characteristics before deep learning. With AI and machine learning, systems will be able to resist new, sophisticated attacks with shifting characteristics [7]. Drones must be built with a collective machine-learning model integrating all data from IoT devices and webcams that can be sent to the MEC to create an algorithm with strong predictive capability. Privacy concerns indicate that if all data are stored on a centralized server, anyone can access the drone data, posing a security risk [8]. AI has grown rapidly and successfully due to the tremendous amount of data available. Federated learning (FL) is a new distributed collaborative AI paradigm developed by Google [9]. It coordinates AI training between multiple clients (such as hospitals, banks, and others) without sharing raw data. It is particularly useful for sensitive data such as patient health records, workplace safety information, and personal banking information that needs to be gathered privately and securely for training and testing [10]. By collaborating with different training nodes, FL allows machine learning algorithms to be decentralized. Each node uses its local data and shares model updates, resulting in a single model trained from its local data without the need for centralized data sharing. By distributing computation to different nodes of MECs, federated learning maintains edge devices’ private data, as there is no need to share it with the core network [11,12].

While FL prevents data leakage induced by raw data [8], it lacks sufficient and transparent procedures to assess contributions and train local models to ensure ongoing, active training. Therefore, UAV networks and blockchain technology will assist FL systems to overcome obstacles and address security and privacy issues associated with MEC networks assisted by UAVs [13,14]. Blockchain provides a ledger technology that enables model decentralization by removing the need for a core network. It provides the ability to gather data models securely from several points or locations [15,16]. The blockchain facilitates uploading and tracking updates, rewards users for training or validating models, and makes updates immutable and secure [11]. As a result of smart contracts, centralized federated learning provides the core network. Training various nodes on different datasets and aggregating or averaging their updates eventually led to a reliable global model. The goal is to obtain a model that minimizes the averaged loss functions of all participants. In addition, the updated global model is added to the blockchain [17]. However, using conventional blockchain and FL methods will not be possible due to the open nature of UAV communication and the MEC paradigm. The proposed framework assumes that UAVs collect data and MEC servers store it in the blockchain. This includes basic data, such as the device name, MAC address and type, and geographic data, such as latitude and longitude, that help MEC servers acquire data. Before data is added to the blockchain, MEC servers verify UAV validity.

In recent years, Deep Convolutional Neural Network (CNN) technology has gained significant traction in image classification tasks, including automatic classification, object detection, and semantic segmentation [18,19]. Increasing GPU computing and open training datasets have led to the rapid development of CNN models such as AlexNet [20], VGGNet [21], ResNet [22], DenseNet [23], and Inception [24], which are increasingly used in conjunction with Fully Convolutional Networks (FCNs). Deep learning requires many labeled samples to complete the corresponding training, and [25] argues that deep learning can be combined with active learning to reduce the human cost of labeling training samples and improve image classification performance [25]. Using aerial images captured with UAVs in Saudi Arabia, this study employs a DenseNet model and architecture combined with a deep-learning network and active learning. This model uses Federated Averaging (FDA) for SGD. The optimization of the loss function is carried out with SGD, as in most federated learning systems. Every MEC runs SGD on its own private data and periodically synchronizes with other MECs by collecting averages and sharing the optimal solution from all the local MEC models to the central network or server to build aggregated global ML models. Homomorphic encryption will be used to protect gradient privacy in a blockchain-based FL scheme and train the local model for the aggregate global model to preserve privacy. The source data is supposed to maintain security and privacy. In this study, DeepAL is employed in federated learning based on blockchain and homomorphic encryption to detect floods from aerial images captured by UAVs.

### 1.1. Motivation for the Study

Smart devices have become essential to our lives. The Internet of Drones (IoD) has become popular in recent years because of its automation, portability, and capability to provide live views of vast areas. It is used in diverse sectors such as the military and healthcare. Researchers have focused on drones because of their advantages, such as mobility. From an IoT perspective, drones play an important role in exchanging information and decision-making. It has become necessary to integrate UAVs and artificial intelligence to facilitate rescues. Drones’ sensors and cameras can access areas that people cannot see.

Using drones can help to save many lives during floods and other catastrophic weather events in places that are difficult for people to reach. IoT devices can be used to collect data on the location and status of people in the affected areas, such as their vital signs, to prioritize rescue efforts. AI has been increasingly utilized in various fields. Among AI techniques, deep learning is a popular and significant approach that relies on neural networks. Deep learning’s success in tasks such as image classification, traffic identification, and self-learning is attributed to the development of related computer hardware and the availability of big data. As a result, deep learning is becoming increasingly important in our intelligent modern society. Blockchain technology can be used to securely store and share data between organizations involved in rescue efforts, such as emergency services and non-governmental organizations (NGOs).

Our motivation for this study is to use the current level of knowledge to develop a system using drones equipped with cameras and sensors to collect data from flooded areas. The data will be sent to a central server where deep-learning algorithms will be used to analyze the data and create a rescue plan. The plan will be sent to relevant organizations involved in the rescue efforts, allowing them to provide aid quickly and efficiently to those in need. Overall, this system has the potential to significantly improve the efficiency and effectiveness of rescue efforts in disaster situations. By utilizing AI, blockchain, and IoT technologies, the system can quickly analyze large amounts of data and provide a comprehensive rescue plan, ultimately saving more lives.

### 1.2. Contributions

This study focuses on using blockchain-based federated learning and homomorphic encryption techniques through UAVs to monitor floods and rescue people affected by flooding, the main contributions of this paper, including:

1. The combination of Federated Learning (FL) and blockchain technology can address security and privacy concerns related to Mobile Edge Computing (MEC) networks supported by Unmanned Aerial Vehicles (UAVs). FL prevents data leakage but lacks clear evaluation procedures for local models. Blockchain enables model decentralization, secure data collection from multiple locations, tracking of updates, incentivizing users for model training or validation, and ensuring secure and immutable updates.
2. Using Paillier Additive Homomorphic Encryption (PHE) to improve data privacy by encrypting model gradients with Homomorphic’s public key before uploading them to the blockchain. This prevents unauthorized access to the information. PHE is preferred over FHE due to its lower computational cost and higher efficiency, making it suitable for industrial applications. Furthermore, stochastic gradient descent (SGD) can be used to share optimal solutions.
3. The off-chain and on-chain storage strategies introduced to address the challenges of transmitting large gradient data on the blockchain, the original large-size weight off-chain (IPFS), and storing the relatively small-size IPFS hash of the original weight on the blockchain.
4. The proposed DeepAL-based classification model aims to improve global learning accuracy while reducing communication costs. DenseNet is a beneficial tool in this model as it addresses the issue of vanishing gradients, reduces parameters, and improves feature propagation. Additionally, pre-trained DenseNet can efficiently process and train binary samples while facilitating better information flow between layers through output concatenation.
5. Finally, we discuss the proposed FDSS model and its limitations for managing floods in remote regions using artificial intelligence and blockchain technology. A framework for implementing these services is being developed, which is one part of our future work.

### 1.3. Structure of the Paper

The paper is structured as follows: Section 2 discusses related works and open problems in the research solution of the presented topic. Blockchain technology, IPFS, and other preliminaries and their use in UAVs to reduce security issues are discussed in Section 3. This section also examines the convergence of the DeepAL-based classifier DenseNet, federated learning, homomorphic encryption, and their benefits. In Section 4, we present how to analyze data obtained from UAV flood monitoring and tracking. Additionally, privacy and security techniques are described. Section 5 explains the proposed method in detail. Section 6 illustrates and discusses the challenges of the proposed method. Section 7 concludes the paper and proposes directions for future research.

Brief notations used in the paper are shown in Table 1.

## 2. Related Works

Researchers have published several studies investigating possible solutions that involve software-based and hardware-based authentication mechanisms. We briefly summarize the surveys in this section and list them in Table 2. In [26], the context-aware mutual authentication protocol for drone networks (CoMAD) is used to authenticate single and multiple drones within a swarm. CoMAD introduced context information to conduct information procedures and reshape swarms by removing or adding swarm members. Context information is classified as secret mission-based data only known to legitimate swarm members. The swarm was managed by a master drone not protected by secure channels or storage. To ensure resilience against collisions, the SHA version 2 or 3 with a minimum 256-bit output size was used when new nodes, for which there was no antecedent context information, asked for authentication. Scyther was used to formally verify and informally test CoMAD’s security performance. ProVerif [27] validated the authentication method’s security, and indicative results were provided to demonstrate its computation, storage, and communication efficiency. Potentially malicious drones can use a valid PKI certificate to penetrate a network, even though it is a typical security method used to authenticate network entities. In [28], a distributed authentication method with low computing requirements was created to overcome this drawback. UAVouch enables drones to be identified before they enter a specific group. In addition to exploiting PKI, this method can also detect abnormal mobility patterns on drones by analyzing their trajectory and position in addition to exploiting the PKI principle. The study examined an impersonation attack inside the cell and a Sybil attack outside the cell involving an armored vehicle and several drones. Based on the analysis results, UAVouch achieved detection accuracy scores of more than 85% using INET and OMNetCC. In [29], a lightweight authentication mechanism for drones and ground stations (GSs) is presented by Secure and Efficient Authentication for Unmanned Aerial Vehicles (SENTINEL). With SENTINEL, resource-constrained drones could be authenticated using user-registered flight session keys, and unauthorized drones could not access the IoD infrastructure without a registered flight session key. Instead of a conventional X.509 certificate, SENTINEL uses a specially designed, lightweight binary certificate format. A prototype was implemented using ECDSA, PBKDF-2, and HMAC-SHA256 to validate SENTINEL’s security. SENTINEL outperformed the Transport Layer Security (TLS) for IoT protocol, executing the authentication process 3.1 times faster. In [30], a simple essential agreement framework for the IoD based on the PKI was proposed, where the public–private key pair generated at the Global Control System (GCS) is dynamically changed for each session. In this framework, GCS, civilian drones, and users were authenticated using a cryptographic hash operation. An authentication process consisted of six phases: basic setup, user registration, drone registration, formation of a key agreement, dynamic addition of drones, and robbery of drones. Moving towards a smart IoD environment, a lightweight privacy-preserving scheme (L-PPS) was proposed in [31], significantly reducing energy consumption and computation effort during encryption and decryption operations. Using random labels, [32] proposed a hash-based scheme for the authentication of drones deployed in large swarms. To generate random labels for the tasks, they used lightweight hash functions SHA-266 and SPONGENT-128 and considered military applications. This authentication scheme was verified as accurate, cost effective, and energy efficient using the OMNeT++ framework for testing delay and throughput in a network-simulation environment. In [33], the possibility of drones communicating with Powerful Intelligence Computer Systems (PICS) or Air-borne Control and Command Platforms (AC2Ps) was discussed as a military application of the IoD. It utilized pairing cryptography to generate the one-way hash functions and the public-private key pairs, and the Computational Diffie-Hellman Problem (CDHP) assisted in key exchange. A confidentiality and data integrity framework were developed based on identity and aggregate signatures. A public channel enabled drones and mobile devices on the ground to authenticate using ECC, symmetric keys, and biometrics in [34]. In addition to shorter keys, ECC allows for faster arithmetic operations and minimal memory requirements. A central server registers drones and devices, which then run to monitor the purposes of specific areas, while users can acquire the data the drones collect. Therefore, ECC is ideal for devices with limited resources, such as UAVs.

Based on the Radio Frequency (RF) features during signal transmission using Orthogonal Frequency-Division Multiplexing (OFDM), there were several RF features to consider, including symbol time, subcarrier spacing, Fast Fourier Transform (FFT) length, Cyclic Prefix (CP) length, detection, and signal power. Aside from SGD optimization, the drones secured the model’s parameters with homomorphic encryption. A dataset containing RF signals from 3000 drones was used to test the proposed authentication scheme, and the results showed that it outperformed other ML-based schemes. Based on the flight behavior of UAVs and data processing, ref. [35] proposed a protocol for real-time UAV identification. By analyzing flight data (e.g., speed, latitude, and longitude), this protocol modeled the behavior of trusted UAVs. The approval or denial of UAVs was based on their legal or illegal behavior using Bayesian learning (Kalman filtering).

Additionally, ref. [36] investigated the ECC with addition and multiplication operations and developed an authentication scheme to mitigate security attacks (spoofing, key impersonation, replay, and password guessing) in Wireless Sensor Networks (WSNs). UAVs serve as mobile sinks, collecting data from sensors. This scheme was found to reduce the time it takes for system registration and provides adequate protection against attacks at low computational cost, based on the performance evaluation. In [37], a method was presented utilizing HECC, a hash function, and an 80-bit key for UAV-assist ITS authentication. This method was implemented and tested using a 5G wireless backhaul network with multi-access edge computing capabilities, involving UAVs equipped with sensors and onboard units (OBUs), roadside units (RSUs), and vehicles that sent event-driven messages to RSUs. It was assumed that network entities had insecure communication links, and the DY model exploited this assumption. Formal security analysis was carried out using the ROR model, the AVISPA tool, and the assumption of an active and passive adversary. This method performed better in terms of computing and communication costs based on informal and comparative security analysis. To authenticate UAV clusters to the control center, ref. [38] proposed a certificateless pairing-free aggregate authentication scheme based on elliptic curves. This method avoids the key escrow problem and achieves certificate management. For the UAVs, the aggregator, and the Command Centre (CMC), it was assumed that partial private keys were generated by the trusted Key Generation Centre (KGC). CMC verified authentication responses from the aggregator, which served as a cluster head. This method requires the consideration of two types of threats to model malicious entities realistically. An extrinsic threat can only change public keys, whereas a KGC could acquire the master secret key.

In [39], an ML-based Smart Drone Controller (SDC) framework was proposed to improve intruder drone detection and optimize resource allocation. Multiple WiFi-connected commercial drones can be operated autonomously and collaboratively without the need for Visual Line-of-Sight (VLOS) connections with this framework by adapting the mathematical algorithm to the underlying scenario. Enough sensor data and adequate processing capabilities are directly related to the practical feasibility of machine-learning solutions. In [40], FL was applied to UAV-based IoT networks, where the UAVs trained the learning model collaboratively and locally. A Deep Neural Network (DNN) based on FL was developed with four hidden layers that could accept and reject drones. UAV swarm deployments are made more secure through a situational-aware authentication scheme presented in [41]. All candidate UAVs within a cluster should be considered for the position of cluster head. Due to the need for a secure selection procedure, we leveraged edge intelligence and Linear Discriminant Analysis (LDA) to thwart spoofing attacks. In urban and rural environments, different scenarios accounting for unreliable communication links were considered because there were varying numbers of unreliable UAVs and communication links. The authentication process was strengthened by using unique cross-layer attributes (e.g., RSSI, PER, and latitude). A simulation study was conducted to verify the accuracy of this authentication method and its low computational overhead, as well as to simulate the cross-layer attribute data.

Practical Byzantine Fault Tolerance [42] was adapted to record trusted and potentially untrusted data on a private blockchain. ACSUD-IoD was experimentally tested and proven to be robust against typical attacks. In [43], multiple entities can be a certificateless and ECC-based authentication method, Secure Lightweight Proven Authenticated Key Agreement (SLPAKA), that can cope with the widely used Canetti Krawczyk adversary model [44], presented in [43]. The communication channel could be entirely affected by a probabilistic polynomial adversary in this model. IoD network components included a Trusted Authority Centre (TAC) that generated keys, MEC devices to assist drones with computation tasks, and a GCS. A Python programming language was used to implement SLPAKA and investigate its energy and computational efficiency. The security was examined informally and formally (via the ProVerif tool), and the algorithm’s advantages were demonstrated authenticated simultaneously in a UAV-based network, and attribute-based voting is used in a Blockchain-enabled Trustworthy UAV Network (BT-UAVN). A blockchain system was used in the BT-UAVN to record and manage transactions to be analyzed in the future. First, blocks were built to validate point-to-point data transfer between UAVs and the controller platform using specialized sensors to gather the required data. Next, the voter was hierarchically classified to identify the UAVs and determine their characteristics. Additionally, two attack scenarios, including those at the facility and those at the communication channel, were examined during real-world, hands-on experiments evaluating BT-UAVN’s security. In [44], a blockchain-based method designed to detect non-eligible UAVs was proposed, called Access Control Scheme for Unauthorized UAV Detection and Mitigation in an IoD Environment. An IoD system was considered where mutual authentication was required between UAVs and GCS using big data analytics and AI-inspired decision-making.

We discussed and compared relevant review and survey papers that outline current trends in IoD node authentication and summarized the findings. In addition, we presented a comprehensive overview and classification of recently proposed software-based authentication solutions and hardware-based authentication implementations.

## 3. Preliminaries

### 3.1. Blockchain Technology

Blockchains are decentralized, distributed databases or ledgers of transactions that are replicated and shared among peer-to-peer communities. Blockchain is based on cryptographic principles where every participant (node) maintains its own copy of the ledger, and a consensus protocol confirms transactions before they are added to the chain. Each blockchain block has a unique hash value. Hash values for each block are iteratively hashed to produce a single value [45]. This makes tampering with recorded data complicated [46]. Its primary purpose is to document, distribute, and immutably store digital information, meaning that entered data is irreversible. A description of blockchain technology appeared as a research project in 1991 (Haber Stuart and Stornetta). Bitcoin was introduced by Satoshi Nakamoto in 2008, who invented a decentralized blockchain implementation. In 2015, a decentralized blockchain platform called Ethereum became essential in blockchain technology because of its ability to securely execute and verify application code, known as ‘smart contracts’. Once conditions are met, the digital contract is automatically executed, ensuring data sharing and trust without requiring third parties to be reliable [47,48].

### 3.2. InterPlanetary File System (IPFS)

Our proposed model uses on/off-chain storage to overcome block storage limitations and to avoid potential increased communication delays and overhead caused by the transmission of large weights. Blockchain systems use IPFS as an off-chain database to store data [49,50]. The IPFS distributed file system enables the sharing of files between computing devices based on peer-to-peer networking [51]. Participants upload their encrypted local model weight to IPFS after each round of local training. The IPFS hash will then be included in a transaction sent to the blockchain’s transaction aggregation so it can be used to retrieve the original data. Once a transaction has been added to a new block, a smart contract blockchain will send a transaction receipt that includes the transaction hash, IPFS hash, and block number [49].

### 3.3. Deep Active Learning

Deep learning and active learning are subfields of machine learning. Deep learning is based on artificial neural networks that extract higher-level features from raw data using multiple hidden layers. Deep learning requires large datasets with annotations for training [52,53]. Image classification uses active learning, which iteratively annotates the most beneficial unlabeled dataset so that the classification error is minimized by each iteration. Thus, active learning may reduce annotation costs when the unlabeled data are plentiful, but labeling is costly [53,54,55]. The combination of active learning and deep learning has recently gained popularity, resulting in DeepAL. The first active DeepAL approach was suggested by [25]. Three metrics were used to select data: least confidence, margin sampling, and entropy. The data selection strategy is applied to deep learning networks. There are two main components to DeepAL, the AL query strategy on the unlabeled dataset and the DL model training method.

Active labeling in deep learning usually begins by initializing the parameters of a DL model or pre-training on labeled data SL0 to perform active labeling. DL features are then extracted from the unlabeled sample set SU. With an unlabeled sample set SU and a la-belled sample set SL, we can extract features through the DL model. A subset of the unlabeled samples is selected from SU in each iteration, where the algorithm labels them and adds them to SL to reduce the DL model’s error. This procedure is repeated until the as-signed label runs out or termination conditions are met.

The DeepAL algorithm uses samples to create a more accurate representation of a dataset that is difficult to classify using the current DL model. An entropy calculation determines the most uncertain unlabeled samples S_i_; i = 1, 2, …, n Therefore, DeepAL generates another label training set S_N_. The entropy of class prediction information is chosen using Equation (1) as described in Algorithm 1 [53,56,57].

(1) $$S_{i}=\arg_{S_{i}}\max-\underset{j}{\sum }p\left(h_{j}^{n}{|S}_{i}\right)\log p\left(h_{j}^{n}{|S}_{i}\right)$$

**Algorithm 1.** DeepAL features extraction for images.Input: *θ*: The initialized parameter of the DL model or pre-trained on the labeled    training set SL0   S_U_: sample set of the unlabeled pool   S_L_: labeled sample set   S_N_: new label training sample set   S_i_: unlabeled samples; i = 1, 2, …, n Output: produce S_N_ ∈ S_L_ must label sample from S_U_ For each S_i_ ∈ S_U_; *i* = 1, 2, …, n Step 1: Pick samples that are difficult for the current DL network to classify. Step 2: Use entropy analysis to select the unlabeled samples with the greatest    degree of uncertainty S_i_ where it has the class prediction information   with the highest entropy by using the following equation:          $S_{i}=arg_{S_{i}}max-\underset{j}{\sum }p\left(h_{j}^{n}|S_{i}\right)\log p\left(h_{j}^{n}|S_{i}\right)$ where $h_{j}^{n}$ is the activation value of the unit j in the upper layer out of N deep learning layers. Step 3: Add label Sn samples to S_L._
  S_L_   ←   S_N_ ∪ S_L_ Step 4: update S_U_  S_U_   ←   S_U_ − S_N_
End for 

### 3.4. DenseNet

CNNs have recently demonstrated exceptional performance in extracting spatial image features [58,59,60]. To fully utilize the network’s potential, DenseNet was introduced in 2017 to solve the network/explosion problem with more layers by reducing gradient vanishing before it reaches the final layer. Thus, dense neural networks can grow deeper while maintaining high efficiency [61]. DenseNet [20] is a state-of-the-art CNN architecture that can recognize visual objects using fewer parameters. The layers of this deep-learning architecture are directly connected, allowing efficient information flow between them [23]. DenseNet architecture addresses this issue by densely connecting all layers. DenseNet aims to improve the architecture flow by connecting every layer to every other layer [62]. CNN can make decisions based on all layers rather than just the top. DenseNet is more advanced and can capture image data on a larger scale than conventional image processing techniques [63].

**Figure 2:**
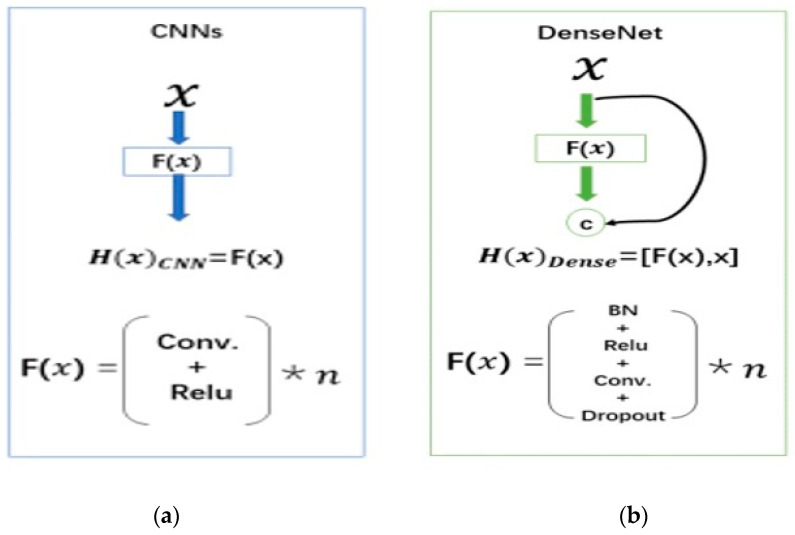
Network architecture:(**a**) conventional CNN and (**b**) DenseNet.

Compared to other pre-training CNN methods, DenseNet offers several advantages, including solving the vanishing gradient problem effectively, reducing the number of parameters, reusing features, and improving feature propagation [64]. Using pre-trained DenseNet, binary samples can be pre-processed and trained quickly. There are fewer parameters in DenseNet than in other CNN models, and features can be concatenated [61]. Using the concatenated (.) attributes, DenseNet combines the output from a prior layer with the output of a subsequent layer and provides a simple communication model that improves information flow between layers. The equation is then again transformed into:

(2) $$X_{L}={\;H}_{L}\left(\left[X_{0},{\;X}_{1},\;\ldots ,X_{L-1}\right]\right)\;\;$$

where $\left[X_{0},{\;X}_{1},\;\ldots ,X_{L-1}\right]$ are the concatenation of the feature maps produced in layers $\left[L_{0},{\;L}_{1},\;\ldots ,L_{L-1}\right]$. Out of the functions, H_L_() represents a non-linear transformation function. This function consists of three major operations, batch normalisation (BN), activation (ReLU) and pooling and convolution (CONV), which are all present in a dense block. The transition layer seeks to increase computation efficiency by minimising the input size. The DenseNet architecture is presented in Figure 2 [61].

### 3.5. Federated Learning (FL)

Federated learning is an optimal approach to protect privacy through edge-based DL. The idea behind FL is to run decentralized ML algorithms without uploading the training set to a central server [8]. Machine learning models are jointly built from data distributed across multiple devices and updated with shared weights to prevent data leakage [12,65]. Federated learning participants can be divided into two classes:

(i) a set of K of participants where $K=k_{1},k_{2\;},\;\ldots ,{\;k}_{n}$ where each participant k_i_ ∈ K has a local dataset D_i_, and
(ii) a central server or central node S.

To reduce the communication rounds, the framework selects a fraction of clients in each iteration instead of all participants. Similar to the learning algorithm of the multilayer perceptron neural network, federated learning aims to minimize the loss function *ℓ*(G) but in a distributed scheme:

(3) $${\;\min}_{G}\ell \left(G\right)=\overset{K}{\underset{k=1}{\sum }}\frac{n_{k}}{n}{\;L}_{k\;}\left(G\right){\;\mathrm{where}\;L}_{k\;}\left(G\right)=\frac{1}{n_{k}}\underset{i\in P_{k}}{\sum }\ell_{i}G)$$

where L_k_(G) is the loss function of kth local client, n_k_ equals the local data size and P_k_ is the set of data indexes whose length is n_k_ (i.e., n_k_ = |P_k_|). Optimising the loss function *ℓ*(G) in federated learning is equivalent to minimising the weighted average of the local loss function L_k_(G).

The suggested system involves a learning model, also called a training model, which is a machine-learning model trained using datasets. In FL, there are two kinds of learning models, the local model, and the global model. Each participant trains their own local model using their own dataset, while the global model is trained collaboratively by all participants. The ultimate goal of the FL process is to obtain a fully trained global model [5].

**Figure 3:**
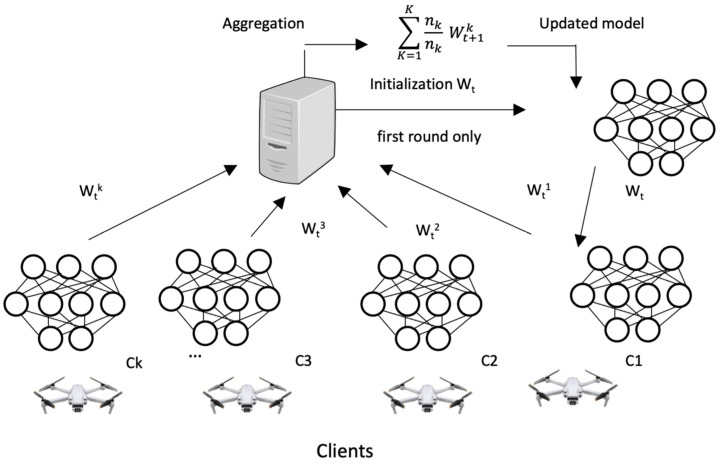
Federated learning architecture.

A federated learning system requires more communication resources than classic centralized learning. Hence, the proposed model is based on FL architecture. FedAvg [9] improves communication efficiency by reducing local training minibatch sizes and increasing local training passes. Global models are created using FedAvg by combining the results of local models. Recently, SGD variants have been used to optimize deep-learning applications almost exclusively [9]. Additionally, DeepAL is used in federated learning, which can reduce communication costs without reducing global learning accuracy. FedAvg pseudo code is presented in Algorithm 2, which builds a federated model with K clients using the FedAvg algorithm [9,54]. Figure 3 illustrates a blockchain-enabled federated learning model for privacy preservation.

Algorithm 2 is split into two sections. The first details the server-side operations and the second outlines the actions taken by each client. W_t_ represents the global model parameters, and the client selection parameter is denoted by C, a random number between 0 and 1 that determines the total fraction of C × K clients permitted to update the shared global model. The number of FL participants is represented by m, W_k_, K, B, E, η, nk, and n denotes the kth local model’s parameters, the total number of clients, the minibatch size, the total number of training iterations, the learning rate, the data size at client k, and the size of the entire dataset, respectively [9].
**Algorithm 2.** Federated learning architecture motivated by FedAvg Section 1: Global trained model Operations on the server side:  Initialize the global model parameters W_t_. for each communication round t = 1, 2, …, do  Select m = C × K, C ∈ (0, 1)   Download W_t_ to each FL participants k.  for each FL participants (k ∈ m) do    Wait Client k for synchronization.   Compute $W_{t}\leftarrow \overset{m}{\underset{k=1}{\sum }}\frac{n_{k}}{n}W^{k}$   Download the federated gradient $W_{t}$  end for end for  Section 2: Operations on the clients’ side (suppose client at k): Update local weights using $W_{t}\;$and η.                       $W^{k}=W_{t}$ B ← (split P_k_ into batches of size B) for each local epoch i from 1 to E do  for batch b ∈ B do                  $W^{k}\leftarrow W^{k}-\eta \;\nabla L_{k}\left(W^{k},\;b\right)\;$  end for end for Return $W^{k}$ to central node.

### 3.6. Homomorphic Encryption (HE)

Federated learning and blockchain-distributed ledgers are used to update the global model. Blockchain gathers data models from various nodes by combining local and global models. Afterward, the smart contract uploads the weights and updates the models. A blockchain-federated learning architecture is proposed for complete decentralization and enhanced security. Furthermore, decentralization increases the model’s accuracy and makes it poisoning-proof [56]. Although FL provides privacy-preserving training with distributed data, adversaries can still reveal sensitive information by sharing weights. Therefore, the suggested system uses homomorphic encryption based on FL. By using homomorphic encryption, it is possible to perform a computation on a ciphertext and obtain an encrypted result identical to the result obtained from a plaintext computation. When transferring intermediates, homomorphic encryption shields the gradient weights effectively. There is no gradient leakage in the proposed scheme. In homomorphic encryption, third parties such as service providers or clouds may compute specific functions on cipher data while preserving the data’s format and function. Additionally, HE is related to mapping for abstract algebra. For instance, suppose that there are two messages, m1, and m2. It would be possible to obtain E(m1+m2) by simply using E(m1) and E(m2) without realizing m1 and m2 as E denote the function of encryption. Equation (4) illustrates this:

D_pr_ (E_pk_ (m_1_) · E_pk_ (m_2_)) = m_1_ + or ∗ m_2_ (4)

D_pr_ indicates decryption using private key pr. D_pr_ indicates the decryption process by using the private key pr. E_pk_ represents the encryption process by using the private key. Encryption is an essential mechanism for saving private and sensitive data. Traditional encryption methods need to decrypt data first to work on the encrypted data. Homeo-morphism was first used in 1978 by R. L. Rivest and M. L. Dertouzos in their research [66]. Homomorphic encryption is an ideal solution for computing without decryption issues. It is composed of four main algorithms: key generation, encryption process, decryption process, and evaluation.

In the first stage, the key generation algorithm generates a public key, P_k_, and a private key, S_k_. In the encryption algorithm, the plaintext M is taken, and then the ciphertext C is produced. In the decryption process, the ciphertext C is decrypted using the private key, S_k_, resulting in the original text M. In the evaluation algorithm with the function F and the block of ciphertexts C_1_, C_2_, C_3_, …, C_n_, the public key is accepted first. The output would be Cf: Cf = eval (P_k_, F, C_1_, C_2_, C_3_, …, C_n_).

**Figure 4:**
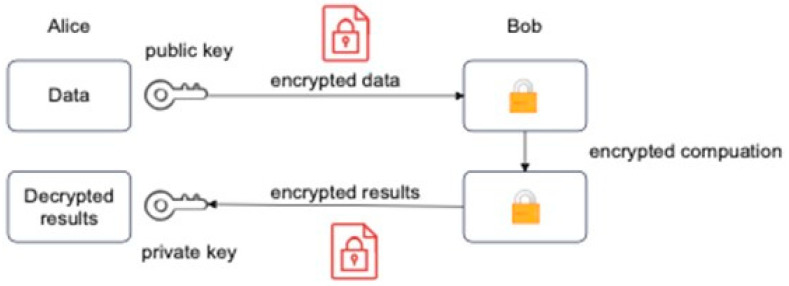
Homomorphic encryption.

According to the operations’ numbers, homomorphic encryption can be classified into three types. Partially homomorphic encryption (PHE) supports one operation; somewhat homomorphic (SMHE) supports two operations and fully homomorphic encryption (FHE) supports unlimited operations [67]. We adopt Paillier Additive PHE [68] in our work for three reasons: (1) In our FL model, when the leader node aggregates all encryption weights, only the addition operation (∑) is needed. (2) It allows unlimited addition operations [67]. (3) It has a lower computational cost and higher efficiency compared to FHE, which can almost meet the needs of the industry. Figure 4 shows the homomorphic encryption process.

Homomorphic encryption offers an additional layer of security, as it allows the computation to be performed directly on a ciphertext to produce an encrypted result that is identical to the result calculated on the plaintext. This encryption scheme effectively protects parameters when transferring intermediate parameters throughout the FL training process, and as such, it has been widely used by numerous FL methods. In addition, the homomorphic encryption scheme ensures that the results of the training process remain confidential and secure, and the encrypted result is only visible to those with the necessary access rights. This level of privacy protection allows organizations to confidently use federated DeepAL active learning for their AL needs, knowing that their data remains secure [69,70,71].

The HE scheme requires one trusted server in the cloud with a client interface for all users compared to other encryption computing schemes. The most well-known HE application is when data owners send their data to cloud servers for processing without trusting the providers. HE enables data owners to encrypt data and send it to the server securely. In addition, homomorphic encryption allows machine-learning computations to be applied to encrypted data. Additionally, it will be possible to derive insights after combining multiple sensitive datasets by preserving privacy after running an encrypted query on a cloud database. In homomorphic encryption, data is converted into an encrypted form for analysis and use. With homomorphic encryption, complex mathematical operations can be performed on ciphertext without compromising encrypted data. Homomorphic encryption eliminates trade-offs between privacy and usability.

### 3.7. Security Analysis

Security is essential in UAVs and must be addressed. UAVs must be controlled by authorized users only. Security challenges need to be addressed when designing a secure framework for specific missions using UAVs. UAVs can cause harm if used maliciously. For example, drones are equipped with sensors and cameras to collect information, including images, videos, and data, which need to be kept securely. If an attacker hijacks a drone’s information, the information can be compromised [72]. We discuss various attacks targeting IoD in the following sections.

#### 3.7.1. Data Confidentiality

Data privacy concerns data that is not shared or made available to anyone other than authorized users. UAV devices collect confidential data, which have to be secured and protected. UAVs take images while in flight, and these should be confidential. Images can compromise people’s privacy and valuable resources. Security methods should consider storing this sensitive information using secure tools to guarantee security. Encryption, authentication, authorisation, and secure device-to-device pairing can increase UAV security.

#### 3.7.2. Data Integrity

Data integrity ensures that unauthorized users have not altered data. UAVs have no on-board driver. Operations are based on data and control signals given by a remote controller. If data integrity is not applied, data manipulation can occur, such as man-in-the-middle attacks. Data integrity should be guaranteed in UAV system design using security tools such as strongly secured authentication and encrypted data sharing.

#### 3.7.3. Authentication and Authorisation

UAV operations need to be controlled using a ground control station. Authentication and authorisation are required to access system resources and services. It is essential to guard against unauthorized access to the remote-controlled system. Encrypted security authorisation and other methods can raise the security level. After authorisation, users need to be authenticated to ensure security and accountability. Virtual private networks (VPNs) can be used to improve UAV authorisation and authentication security.

#### 3.7.4. Data Fabrication

The false representation of data during sending and receiving can obstruct UAVs’ flight operations. Data fabrication can affect the ground controller and cause problems with UAV control. Fake information can be delivered by intruders from UAV sensors to the controller. Encrypted data-sharing methods and authorized access can decrease the level of data fabrication.

#### 3.7.5. SkyJack Attack

A SkyJack attack is a cyber threat in which an intruder UAV hijacks nearby drones within a wireless perimeter. The intruder finds the MAC address of the neighbouring UAVs within the Wi-Fi perimeter and disconnects the affected UAVs from the control station. The intruder then gains access to and control of the victim UAVs.

#### 3.7.6. False Data Injection

False data injection can destroy system performance. It provides incorrect data, such as inaccurate weather forecasts that mislead farmers. This can lead them to make misinformed decisions, causing financial losses.

#### 3.7.7. Denial of Service (DoS) Attacks

Denial of Service attacks disturb links between UAVs and the ground controller. The attacker floods the link with false data packets, so the link is busy and cannot receive the UAVs’ useful data. An intruder can do this easily if the link is not encrypted.

#### 3.7.8. Wireless Interface Attack

UAVs use many wireless interfaces. Weak security protocols (WEP and WPA) in the Wi-Fi access control in this type of system are vulnerable to attacks.

#### 3.7.9. Tampering Attacks

Attackers can edit unauthorized UAV channels. Data can be tampered with and intercepted. Data should be protected in the channel, which will prevent attackers from intercepting packets and tampering with the destination address.

## 4. Secure Sharing Gradient Model Process

In the proposed model, blockchain-enabled secure FL broadcasts collect and share the encrypted weight with other participants via homomorphic encryption [73] so that attackers cannot infer information from the weight. Three phases are proposed [5,56,74,75].

**First** **phase: Learning participants.**

Every learning participant implements the following steps.

(0) Initialisation: Every participant agrees on a new FL task. The blockchain’s central node will invoke a smart contract using inputs from agreed-upon initialisation parameters (e.g., learning rate *η* and gradient *W_init_* for the first round). Then, a public key pair (*p*_1_, *p*_2_) for homomorphic encryption will be distributed to all participants. We assume that the optimisation of the loss function occurs through a simple SGD algorithm.

(1) Local model training: Each participant creates their own local models *M_local_* with weights *W_init_*. Each participant will decrypt the weight of the global model and utilize their local dataset to carry out the DL training process based on SGD. There are two primary stages to the training process. The first calculates the difference between the model’s predicted value and the training dataset’s actual value to determine the sum of the square’s modeling error E(G). The second minimizes E(G) using a derivative function to obtain the model gradient updates.

(2) Initial global model training with label data: This is for all participants using the public key pair (*p*_1_, *p*_2_). For any gradient, they randomly choose a number *r* that satisfies 0 *< r < p*_1_ and compute the ciphertext$\;Enc_{p1}(W_{local\_K})\leftarrow Enc(W_{global})$ only for the first time. The encrypted weight of the global model is shared with all clients.

Go to phase 3.

Second phase: Share the gradient for the aggregation model in the blockchain.

(1) Uploading encrypted weight: After completing the DL process, all participants send transactions to the blockchain network to upload the encrypted weight of the local model$\;Enc_{p1}(W_{local\_K})$ to the blockchain network by sending the transaction for the aggregation model.

(2) Aggregation and decryption: Upload $Enc_{p1}(W_{local\_K})$ after m iteration to the central node in the blockchain to perform the aggregation function, such as FedAvg SGD to calculate the average all encrypted weight of local model $Aver\left(Enc_{p1}(W_{loca.k}\right))$ from a transaction that exists in the transaction pool.

(5) $$\begin{array}{c} Aver\left(\;Enc_{p1}(W_{loca.k}\right))\\ =\frac{1}{K}\;⊗\left(\;Enc_{p1}(W_{loca.k1}\right)\\ ⊗\;\left(\;Enc_{p1}(W_{loca.k2}\right)\ldots \left(\;Enc_{p1}(W_{loca.kn}\right))\; \end{array}$$

(6) $$W_{loca\_k}\;\;\leftarrow Aver\left(Enc_{p1}(W_{loca.k}\right))$$

(7) $$\;\;\;W_{agg\_F\left(i\right)}\leftarrow \;Dec_{p}{}_{2}(W_{local_{k}})$$

then decrypts the aggregation gradient with its own private key.

(3) Broadcast global gradients: Download the federated weight of the global model $W_{agg\_F\left(i\right)}\;$to other replica nodes that are responsible for block consensus.

(4) Transactions committee and create new block: The transactions are committed when all the nodes across the network agree where the transactions are assessed on the blockchain by a set of smart contract rules. The rules for the agreement on a particular block are based on consensus.

Replica nodes of blockchain networks send commit packages to other nodes. If valid, the transactions are committed on the blockchain by recording it in a traditional database. After receiving enough commit packages for validation, the central node can generate a new block involving the transaction of$\;W_{agg\_F\left(i\right)}$.

Third phase: Broadcasting Global Weight.

(1) Updating local model: The central node broadcasting weight of the global model $W_{global}$ and the learning rate η for all participants to update their local model and execute the DL training process based on SGD was downloaded from the latest block$\;W_{global}$. Then, it saves it as an unencrypted local model as follows:
  (8) $${\left(W\right)}_{\mathrm{agg}\_F\left(i\right)}\leftarrow {\;\mathrm{Dec}}_{p}{}_{2}(W_{\mathrm{global}})$$
(2) Download the federated gradient$\;W_{agg\_F\left(i\right)}$
(3) For an SGD-based optimisation: The updated local weights using $W_{agg\_F\left(i\right)}\;$and learning rate η are calculated as follows:

For each batch b ∈ B the minibatch size indicates the amount of the local data used per each participant node.

(9) $${\;W}_{\mathrm{local}\_K}\leftarrow W_{\mathrm{agg}\_F\left(i\right)}\;$$

(10) $$W_{\mathrm{local}\_K}\leftarrow W_{\mathrm{local}\_K}-\eta \;\nabla l_{k}\left(W_{\mathrm{local}\_K},\;b\right)$$

(4) Return *W_local_k_* to central node.
(5) Repeat the local model updates: Repeat the steps in the second and third phases until the iteration number of training reaches the upper limit to produce the global model, which means the loop-end condition is when the gradient is$\;W_{agg\_F\left(i\right)}=0$.

As part of active learning operations, clients train a classifier on a seed (small, manually labeled samples) in the first round. After that, the model forecasts labels for unlabeled samples and adds them to the seed based on the parameters defined in the sampling strategy. This process obtains the most relevant sample from the pool each time. The samples will be retrieved from the pool until a stop condition is reached. The maximum number of iterations is critical because, after a certain number of iterations, the relevance of the chosen sample will decrease, forcing DeepAL to include irrelevant samples. Once the model precision has reached a constant level, the maximum number of query iterations is used as the stop criteria. Using a pre-trained deep-learning model called Dense-Net, the first step of the process extracts features from the input images [20]. DenseNet is used to extract object-level characteristics from input images using a dataset trained on image classification. The pseudo-code of server model training with labeled data and encrypted model aggregation is shown in Algorithm 3, and active learning using unlabeled data in each client is shown in Algorithm 4.
**Algorithm 3.** Server model training with labeled data and encrypted model aggregation **Input:** *L:* Labeled example set *P*_1_: public key *K:* the number of clients W_local_k_: weight of client model W_local_k:_ weight of client model **Output:** Aggregated weight of global model *W_agg_F(i);_ i = 1*, *2*, *…* **Process 1: Initial global model training with labeled data** **1.** Foreach h ∈ W_local_k_; K = 1, 2, …, k do **2.** h ← W_global_ **3.** h**.** *f it*(*L*) **4. foreach**
*layer* ∈ *h*
**do** **5.** W_global_  ←  Encr_p1_(layer, weights) //encrypted weight of global model **6.**
*send*_*to*_*client Wglobal* **Process 2: Encrypted model aggregation** **7.***W_agg_F(i)_*← W_global_ // initial weight aggregation **8. foreach** h ∈ W_local_k_; K = 1, 2, …, k **do** **9. foreach** [*row*] ∈ *h*
**do**  **10.** [*row*] ← *layer*.*weight* //get the core row for layer **11.***W_agg_F(i)_* = *W_agg_F(i)_* ⊕ [*row*] //homomorphic addition **12. foreach** [*row*] ∈ *W_agg_F(i)_* do* **13**.[*row*] ← [*row*] ⊗ 1/K //homomorphic multiplication **14. Return *W_agg_F(i)_***
**Algorithm 4:** Active learning using unlabeled data in each client**Input:** *U*: Unlabeled example set *P*_2_: private key W_global_  ←  Encr_p1_(layer, weights): encrypted weight of global model **Output:** Encrypted weight of local model W_local_k_
 **Step 1: decrypt global model and save it as unencrypted local model**
 **1.** Foreach h ∈ W_local_k_; K = 1,2, ….k do **2.** h ← W_global_
 **3. foreach** layer ∈ h **do**
 **4.** [row] ← layer.weight //get the core row for layer **5. foreach** layer ∈ h_un_ **do**  **6.** h_un_K_ ← Dec_p2_([row])_global_
**// save** unencrypted local model. **Step 2: execute AL training and updated local model ** **7.            While** (!Query Stop Criteria) **do** **8.**batch_samples←  predict(U) // predict unlabeled instances. **9.** h_up_K_ = active_learning(h_un_K_, batch_samples) // updated local model **10. Step 3: encrypt the weight of local model and shares it to the server.**  **11. foreach** layer ∈ h_up_K_  **do**
 **12.** W_local_k_   ← Encr_p1_(layer, weight) of h_up_K_ **13. Return** W_local_k_
**to the server.**

The proposed method consists of six main entities:

1. IoT devices or users: The IoT devices send requests to the cloud server to start the mission. Only authenticated users can start the UAV device.
2. Secure server: Upon receiving a request from the IoT device, the secure server will check the IoT device ID in a secure database in the secure server. The secure server will assign a drone to the IoT device if it is authenticated. Information about the assigned drone, such as its drone ID and timestamp, will be encrypted and sent to the IoT device.
3. UAV device: A UAV receives requests from a secure server. These devices are composed of actuators and sensors. They enable tasks to be performed and sense the environment. UAV devices contain sensors to collect data such as images, altitude, longitude, RGB images, speed, and battery level, as well as IoT sensors in dams and torrents.
4. MEC server: The MEC server collects data (images, IoT sensor data) from various UAVs and trains them as local models. The MEC server will synchronize all communications between UAVs and IoT sensors. Once the mission is complete, the MEC server will send the encrypted gradients (results from different nodes training on different datasets) to the core network as a blockchain.
5. IoT sensors: The sensors will be installed where floods may occur, such as areas prone to torrents, and will synchronize with the MEC devices.
6. Core network: Local models aggregate their updates into a global model that is added to the blockchain. A secure place to keep sensitive data is needed to store updated blockchains. The server is encrypted using two-factor authentication to ensure high levels of integrity and confidentiality. It is important to note that not all data will be stored as a blockchain. Mission information will be stored in blockchain format at the core network to maintain secrecy and privacy. To decrease the core network load, other inessential data is stored in an off-chain database, such as flight information, longitude, altitude, and battery level.

## 5. The Proposed Method of Negotiations

**Figure 5:**
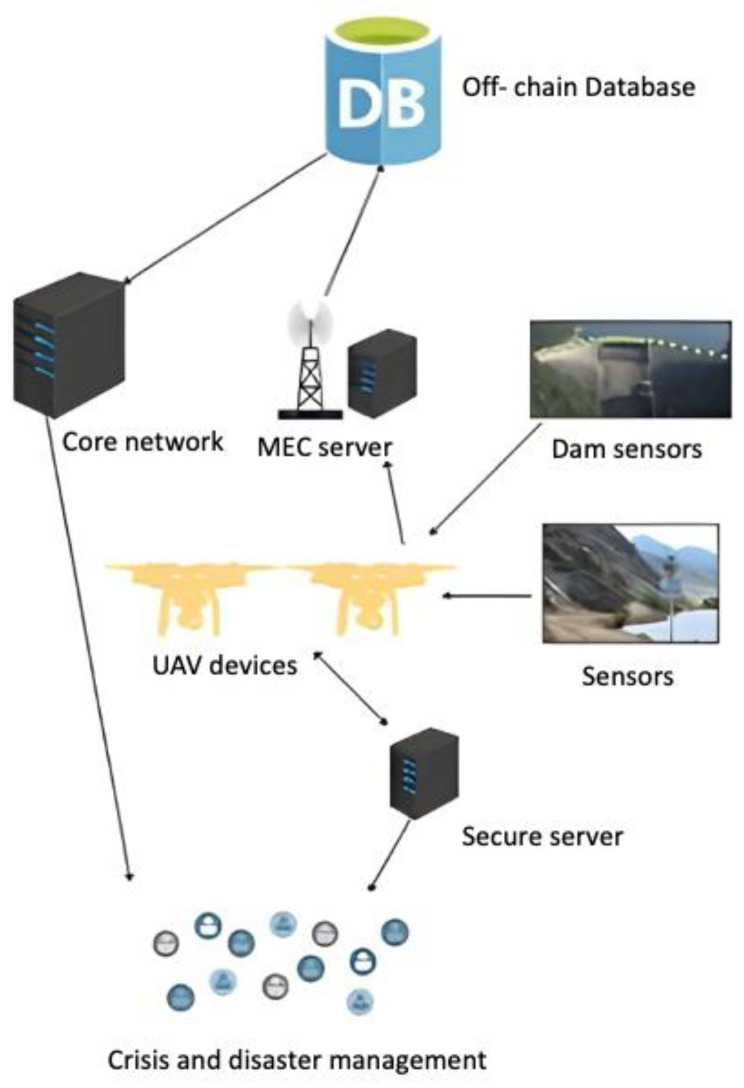
The proposed FDSS method.

An FDSS system ensures that IoT devices are validated before they send information or start a new mission. The proposed method is illustrated in Figure 5, where the IoT device Dv requests a new mission from the secure server Ss. The request includes the IoT device ID, timestamp, and hash to ensure privacy. Ss checks the IoT device and identifies its type: known or unknown. An IoT device that is known to the system means it is registered. An IoT device that is unknown means it has not been registered yet. When the Dv is unknown, Ss sends that kind of unknown device as suspicious S.

h(Request (TS|Dv) Dv: Ss

MAC addresses for known IoT devices are encrypted and sent along with the authentication conformation for the IoT device, including the timestamp and nonce.

E_n_ (*h*|*n*| MAC_A_) ^ N_o_ S: D_s_

The secure server will assign an available drone to the IoT device, and the drone ID and timestamp will be delivered to the IoT device. Using the public key, private key, and XOR filter, the secure server will hash and encrypt the information as follows:

XOR_f_ [*h* (E_n_ (P_u_ (P_v_|TS|*n*)))) ] S_s_: D_s_

Once the UAV device and the IoT device start a mission, the IoT device sends commands to the UAV device. As soon as the UAV device receives the command, the MEC server syncs this data.

*H* (L_a&l_|V|TS) D_v_: U_d_

Data analysis will take place in the MEC server. For example, pictures taken by sensors and UAVs will be maintained. Pictures that reflect floods will be determined using an AI algorithm. Sensors that are in torrents will indicate floods. All this data will be processed on the MEC server. The mission information will be stored in the core server as a blockchain. Other information relevant to flight details and battery life will be saved in an off-chain database. UAVs are required to meet many requirements and specifications when detecting floods in remote and hard-to-reach regions using drones. In real time and in a specific place, the UAV can detect floods, locate people in danger, and deliver emergency supplies. Drones help manage crises and disasters.

**Figure 6:**
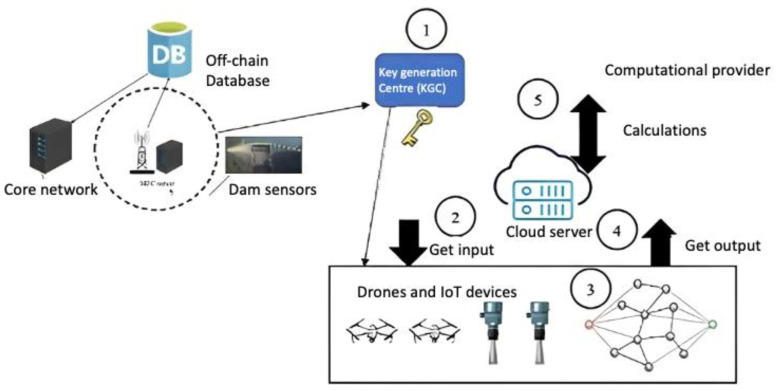
The homomorphic encryption location in the proposed method.

Figure 6 shows federated learning within the MEC server and the use of homomorphic encryption.

Horizontal federated learning is assumed in the proposed model. The main difference between horizontal and vertical federated learning is that the latter uses the same datasets across all devices. However, vertical federated learning uses multiple datasets to train the global model. Key generated centres are considered trusted parties to perform authentication processes and generate key pairs. For each iteration, the cloud server selects UAVs and IoTs based on the trained datasets. A computational provider directly communicates with the computational provider.

### Applications of the Proposed Method

**Figure 7:**
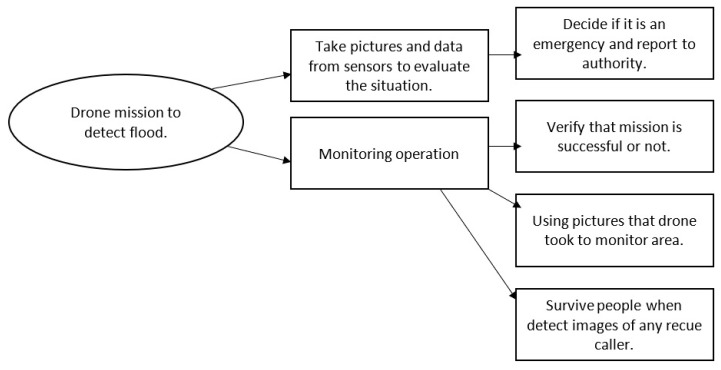
Drone mission to detect flood.

**Figure 8:**
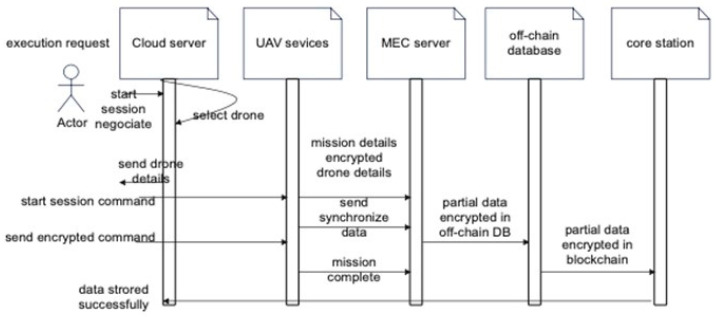
The sequence diagram of the proposed method.

The blockchain can preserve data transmitted via drones and ensure their confidentiality. Data secrecy and privacy would be guaranteed by cryptography in the proposed method, which combines hash functions and asymmetric keys. Timestamps and nonces ensure that data will be available and accurate for a specific period. Drone and IoT device negotiations are synchronized regularly to the core network or blockchain. Alternatively, some detailed information that overloads the core network will be stored in an off-chain database. Figure 7 shows the drone mission for detecting floods, and Figure 8 shows the sequence detection method.

**Figure 9:**
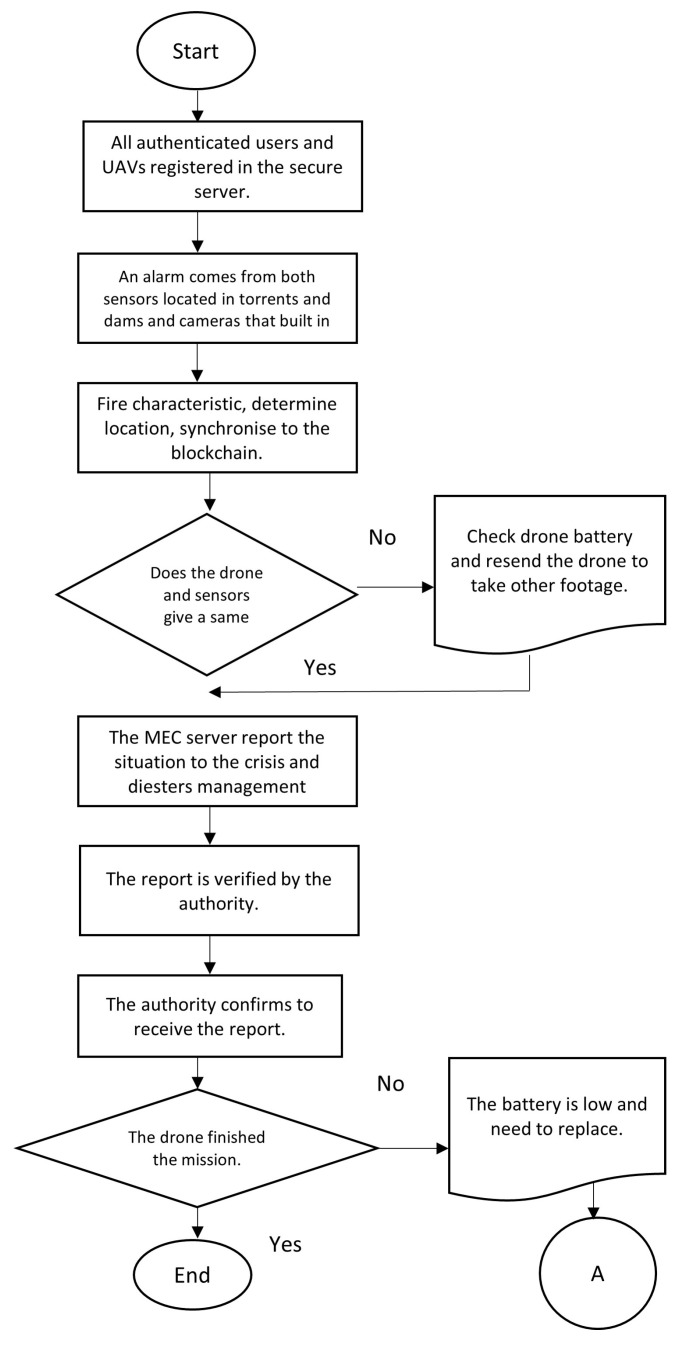

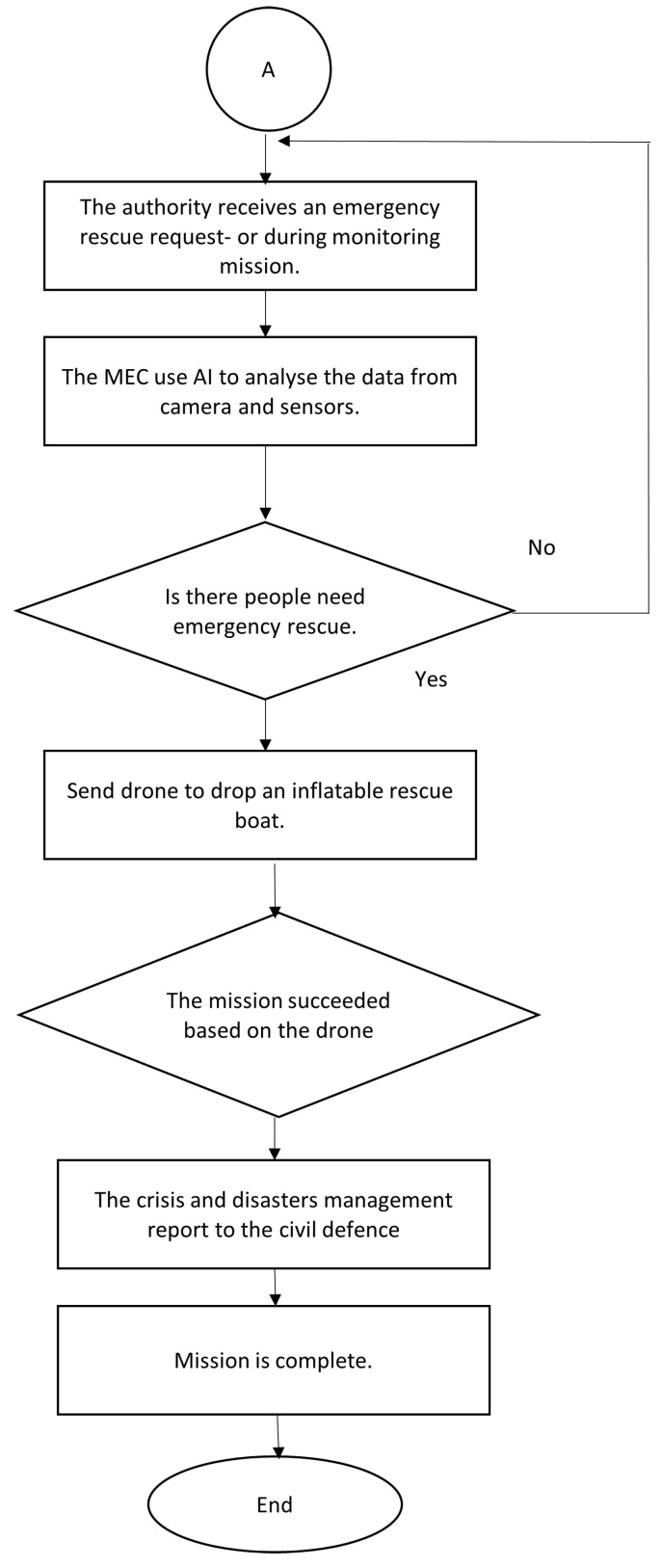
The algorithm of the proposed method.

**Figure 10:**
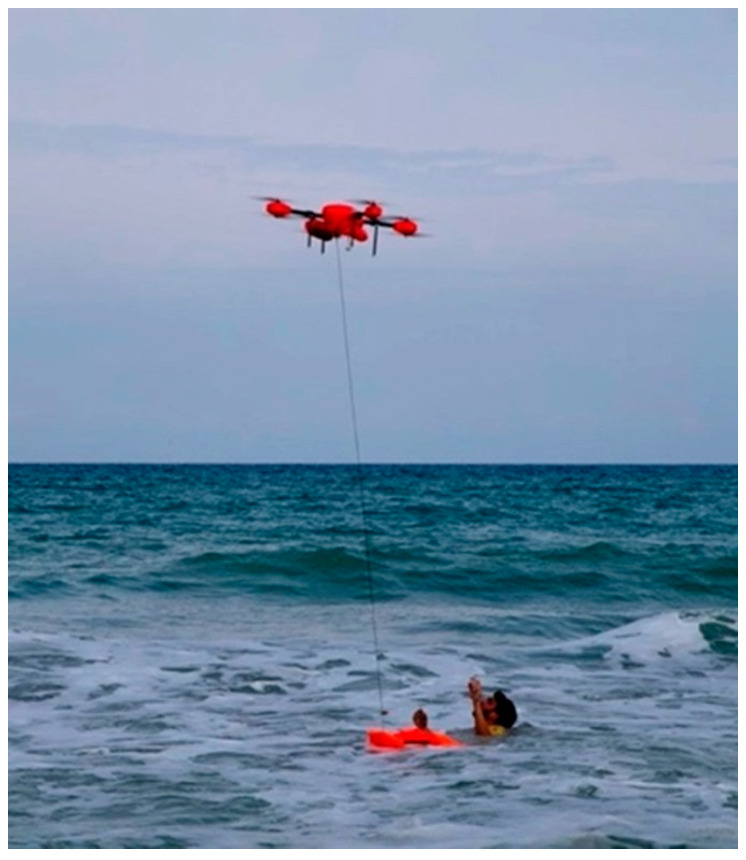
Drone that holds an inflatable rescue boat.

The proposed method may be used in the following applications:

- Drones to detect and monitor floods: A proposed method to detect and monitor floods using drones is shown as an algorithm in Figure 9. Drones with high-resolution cameras are suggested for this proposed method. Data collected from drones report the status in a specific area and give an overview of the situation.
- Drones to rescue people: Drone footage can be used to rescue individuals. Once a picture detects thermal images and the MEC server analyzes them and confirms the results with the local sensors, the emergency alert is raised. As shown in Figure 10, there is a place in the drone to hold the compressed inflatable rescue boat.

The rescue and deliver inflatable boat flowchart shown in Figure 8 includes the following:

1. Civil defence receives the report, or the MEC server raises the alarm.
2. The mission starts, and the drone flies to the desired location.
3. The MEC server reports the emergency rescue mission if thermal images are detected.
4. Drones can provide inflatable rescue boats and the mission’s progress.

The drone coordinates in the area and maintains its trajectory to find flood victims or rescue people. A drone returns to its base once the inflatable rescue boat is used. The rescue mission is over once this has been completed.

## 6. Discussion and Challenges

Drones are now used to solve many problems due to recent significant advances in drone technology. Drones can monitor torrents or cold environments, which can be dangerous. This study proposes a novel method to detect floods using a secure blockchain. To avoid overloading the core network with unnecessary data and real-time images, water levels and wind speed sensors will be stored in off-chain database. Off-chain databases will store inessential data such as flight details, longitude, and latitude. IPFS is used as an off-chain database in the proposed method. The data is large and needs to be stored in a blockchain. As a distributed peer-to-peer database, Orbit DB is used with IPFS. Drones can capture many images during missions, requiring high memory capacity. Only images detected as floods will be stored in the core network, and other images will be stored in the off-chain database.

To illustrate the mechanisms of the proposed method, suppose the drone takes images that shown flood and sensors detect high level of water. The data will be processed and trained using deep active learning and the dataset is created. Using entropy to class predication information as it is explained in algorithm 1. DenseNet is used to develop the accuracy that is caused by vanishing gradients and improve the feature propagation. The pretrained DenseNet helps to process and even train binary samples and obtain optimal information flow between layers. Moreover, FL is used to enhance the security and privacy as it enables decentralized ML without uploading the whole datasets. In addition, the usage of SGD to improve optimization in ML applications. FL takes the minibatch to train and calculate gradients, and weight update the datasets per epoch as it is shown in Algorithm 2. In order to improve the security and ensure that intruders cannot compromize the sensitive data, homomorphic encryption based on FL. The Paillier Additive Homomorphic Encryption (PHE) is used in the proposed method for three main reasons. First, in the FL model, the leader node aggregates the whole encryption weights, but only the addition operation (∑) is required. Second, it enables unlimited addition operations to be used. Third, it has less computational cost and higher efficiency than FHE. The usage of PHE to enhance the confidentiality level.

The combination of the previous technologies is discussed as secure sharing gradients model process in Section 4 and the pseudo code of the system is presented in Algorithm 3 and 4.

In the mission area, blockchain controls the drone’s performance and enhances collaboration. Drones can deliver inflatable rescue boats in real-time to flooded areas, develop the emergency response to handle flooding, and help civil defence respond to save people in danger. Governing and managing the people who implement the code is also essential. Drone sensors and cameras can collect massive amounts of data, but the battery constraints result in suboptimal results. Hence, it is important to put the drone in sleep mode in between missions. UAV images help to estimate flood shores and rescue emergencies.

However, the proposed method has some limitations. First, drones’ usage without permission may be restricted. Second, weather conditions may affect drone quality. Considering the wind velocity in autumn and winter, missions will be most successful in the morning as lightning will increase the clarity of the images.

## 7. Conclusions and Future Work

Flood mitigation, warning, evacuation, and recovery strategies are continually funded and implemented by the government. Utilising emerging technologies, these strategies continue to evolve flood prediction methodology. Drones are becoming an increasingly important part of flood management before, during, and after flood events. This work makes significant contributions towards establishing the hardware and software technologies required to deploy secure and decentralized Unmanned Aerial Vehicles in flood monitoring. The proposed system can provide secure flood management data collection and flood tracking over time. Drones can be used to monitor operations to avoid undesirable results [76]. They can be used to obtain aerial photography for disaster management investigations. By providing detailed descriptions of equipment, aerial pictures of operations assist in understanding scenes.

Drones can also be deployed quickly during safe weather breaks that would otherwise ground aircraft. As manned aircraft do not have to worry about collecting footage while searching for and rescuing people stranded by flooding, drones can also capture footage of flood events. During flood events, satellites are often unable to capture useful images due to cloud cover. In the aftermath of floods, assessing the extent of the damage becomes a challenge. Identifying priority areas for cleaning and repair will help communities to recover. Using drones to survey receding water can often be cheaper and easier than traditional plane or helicopter surveys. Drones can also map damage more precisely than satellites, enabling large-scale damage assessments. Decision-makers need accurate flood damage assessments to determine where and how much support is required. Since UAVs can operate in remote and difficult-to-access areas, they can facilitate enhanced situational awareness for emergency response and disaster management applications [77]. Additionally, by using deep learning and an embedded platform, autonomous UAVs can monitor a disaster-stricken area, analyze images in real-time and alert when various calamities occur, such as collapsed buildings, floods, or fires, so their effects on the environment and people can be mitigated faster. Blockchain technology enables data transparency, immutability, and decentralisation.

In this paper, we introduce a drone application that uses blockchain to manage flooding in remote regions safely and in real-time. The framework can be helpful in missions based on both blockchain and IPFS. The proposed architecture of system nodes makes the process more secure by preventing information from being manipulated and enhancing the data analysis capability within the management system. In a blockchain network, the text data is recorded as part of the transaction information that is recorded during transactions. In addition, a visualisation platform allows access to transaction data, making it easier for operators to supervise their operations. We propose a scheme that improves the FL system performance by using DeepAL to select the optimal edge nodes and integrating the learned model parameters into a blockchain-based FL scheme to enhance the reliability and security of the FL system. This method is combined with modern cryptography techniques, such as homomorphic encryption, to achieve a high level of privacy and security capabilities. This paper presents frameworks to deliver and monitor data in the blockchain to handle data. The frameworks facilitate the flood management process in remote areas prone to flooding. Flexible deployment and robust information analysis can assist in flood insurance monitoring and mission tracking by providing a reliable information base.

However, there are concerns and challenges regarding the large volume of imagery data and storing it in blockchain networks. In future work, these challenges should be addressed. Based on the literature reviewed, we must consider how difficult it may be for an operator to handle the UAV when weather conditions are not as favourable as when the tests were conducted. A regular UAV should not be used when it is raining, but it would be necessary to study how wind or rapid temperature changes can affect flight and up to what speed the UAV can withstand sudden wind gusts. IPFS provides an additional layer of protection against data tampering but requires many peers to work effectively. It is possible to solve this problem in the short term (caching files with tools such as the Public Gateway Cacher). Still, the most suitable long-term solution is to increase the number of peers so files can be accessed more easily, which is difficult with a single node.

Typically, this application domain does not allow popular and shareable images, videos, and files relating to being uploaded to research and crisis areas, which are less engaging than other multimedia content, such as music and videos.

## Figures and Tables

**Table 1:** Brief notations.

Notation	Description	Notation	Description
N	Nonce	N_o_	Normal
TS	Time Stamp	E_n_	Encryption
D_v_	IoT Device	D_e_	Decryption
M_s_	MEC server	P_u_	Public key
U_d_	UAV device	P_v_	Private key
S_s_	Secure server	*H*	Hash
V	Validation process	*N*	Nonce
L_a&l_	latitude and longitude	XOR_f_	XOR filter
MAC_A_	MAC address	FDA	Federated Averaging
S	Suspicious	SGD	stochastic gradient descent
M	Malicious		

**Table 2:** An overview of recent research on the IoD’s software-based authentication mechanisms.

Reference and Name	Advantages and Achieved Performance	Type of Threat Model	Network Environment	Limitations
[26] CoMAD	Security layer and obscurity are increased	Extended DY Model	FANET	CoMAD makes use of a centralized architecture and not tested with decentralized
[28] UAVouch	A high level of detection accuracy with an acceptable overhead	Informal threat Model	FANET	RSA key size can be a problem for hardware-limited systems
[29] SENTINEL	A low computational overhead, reduced traffic, and an efficient execution	Dolev-Yao (DY) Model	Typical IoD	-
[30] PKI-based authentication protocol	An efficient and robust system	Informal threat Model	Typical IoD	The communication cost of the proposed scheme is slightly Different
[31] L-PPS	Energy efficiency, computation cost and robustness	Informal threat Model	UAV-based IoT	Less computation and communication costs
[32] A Random Label and Lightweight Hash-Based Security Authentication	Throughput and delay have been increased	Informal threat Model	Large-scale UAV Swarm	-
[33] Identity and Aggregate Signature-Based Authentication Protocol	Complexity is low, and communication and computation are low	Informal threat Model	IoD for military Scenarios	-
[34] An ECC-Based Authentication Scheme	Security vs efficiency trade-off	eCK adversary model	Typical IoD	-
[35] Intelligent UAV Identity Authentication and Safety Supervision	Modeling complexity is low, while accuracy is good	-	UAV-based Network	-
[36] A secure authentication scheme framework	Cost-effective computation	Informal threat Model	Wireless Sensor Network (WSN)	Limited resources and energy available
[37] A Provable and Privacy-Preserving Authentication Scheme	Low computation and communication costs, small key size and enhanced secrecy are all benefits	DY model	UAV-enabled IntelligentTransportation Systems (ITS)	-
[38] pairing-free authentication scheme (CLAS)	The ability to be unforgeable and practical	Informal threat model	UAV-based Network	-
[39] SDC framework prototype	Detection of drone intrusions	-	UAV swarm	-
[40] a federated learning-based drone authentication model	An excellent accuracy rate	Informal threat Model	UAV-based IoT	-
[41] CH safeguarding mechanism	The system is accurate and low in computational overhead	Informal threat Model	UAV swarm	Low computational complexity in the UAV network
[42] a novel blockchain-based technique	An efficient and secure peer-to-peer network	Informal threat Model	UAV-based Network	-
[43] (SLPAKA) technique for IoD deployment	Cost-effective computation and communication, low energy consumption	Canetti– Krawczyk (CK) Model	Typical IoD	Lower energy consumption and computational time
[44] ACSUD-IoD	Communication and computation overhead is low, and robustness is high	DY and CK Models	Typical IoD	-

## Data Availability

Not applicable.
